# Validation of Dairy Cow Bodyweight Prediction Using Traits Easily Recorded by Dairy Herd Improvement Organizations and Its Potential Improvement Using Feature Selection Algorithms

**DOI:** 10.3390/ani11051288

**Published:** 2021-04-30

**Authors:** Anthony Tedde, Clément Grelet, Phuong N. Ho, Jennie E. Pryce, Dagnachew Hailemariam, Zhiquan Wang, Graham Plastow, Nicolas Gengler, Yves Brostaux, Eric Froidmont, Frédéric Dehareng, Carlo Bertozzi, Mark A. Crowe, Isabelle Dufrasne, GplusE Consortium Group, Hélène Soyeurt

**Affiliations:** 1 AGROBIOCHEM Department, Research and Teaching Centre (TERRA), Gembloux Agro-Bio Tech, University of Liège, 5030 Gembloux, Belgium; Nicolas.Gengler@uliege.be (N.G.); Y.Brostaux@uliege.be (Y.B.); hsoyeurt@uliege.be (H.S.); 2 National Funds for Scientific Research, 1000 Brussels, Belgium; 3 Walloon Agricultural Research Center (CRA-W), 5030 Gembloux, Belgium; c.grelet@cra.wallonie.be (C.G.); e.froidmont@cra.wallonie.be (E.F.); f.dehareng@cra.wallonie.be (F.D.); 4 Agriculture Victoria Research, Centre for AgriBioscience, AgriBio, Bundoora, VIC 3083, Australia; phuong.ho@agriculture.vic.gov.au (P.N.H.); jennie.pryce@agriculture.vic.gov.au (J.E.P.); 5 School of Applied Systems Biology, La Trobe University, 5 Ring Road, Bundoora, VIC 3083, Australia; 6 Department of Agricultural, Food and Nutritional Science, University of Alberta, Edmonton, AB T6G 2P5, Canada; hailemar@ualberta.ca (D.H.); zhiquan.wang@ualberta.ca (Z.W.); plastow@ualberta.ca (G.P.); 7 Walloon Breeding Association, 5590 Ciney, Belgium; cbertozzi@awenet.be; 8 UCD School of Veterinary Medicine, University College Dublin, D04 V1W8 Dublin, Ireland; mark.crowe@ucd.ie; 9 Faculty of Veterinary Medicine, University of Liège, Quartier Vallée 2, 4000 Liège, Belgium; isabelle.dufrasne@uliege.be

**Keywords:** dairy cow bodyweight, partial least square, feature selection, dimensionality reduction, machine learning, dairy cows, mid infrared spectra

## Abstract

**Simple Summary:**

First, the current work consisted of validating the feasibility of large-scale dairy cow bodyweight prediction from models involving the day in milk, milk yield, parity, and milk mid-infrared spectrum. Second, it aimed to improve the accuracy of predictive models by using feature selection algorithms to decrease the number of predictors to limit overfitting. The models, using accessible and low-cost measurements, provided highly reproducible predictions. These could be easily obtained on an individual basis throughout a cow’s productive life by dairy herd improvement organizations, thus providing potentially relevant information for the dairy farmer at three levels: economics (reproductive performance), animal welfare (disease detection), and environment (methane production).

**Abstract:**

Knowing the body weight (BW) of a cow at a specific moment or measuring its changes through time is of interest for management purposes. The current work aimed to validate the feasibility of predicting BW using the day in milk, parity, milk yield, and milk mid-infrared (MIR) spectrum from a multiple-country dataset and reduce the number of predictors to limit the risk of over-fitting and potentially improve its accuracy. The BW modeling procedure involved feature selections and herd-independent validation in identifying the most interesting subsets of predictors and then external validation of the models. From 1849 records collected in 9 herds from 360 Holstein cows, the best performing models achieved a root mean square error (RMSE) for the herd-independent validation between 52 ± 2.34 kg to 56 ± 3.16 kg, including from 5 to 62 predictors. Among these models, three performed remarkably well in external validation using an independent dataset (N = 4067), resulting in RMSE ranging from 52 to 56 kg. The results suggest that multiple optimal BW predictive models coexist due to the high correlations between adjacent spectral points.

## 1. Introduction

Knowing the live bodyweight (BW) of a dairy cow is an important source of information related to farm management [1], disease detection [2], quantification of dry matter intake [3], estimation of methane production [4,5], or even assessing the reproductive performance of a given cow through its BW changes [6]. Capturing BW can be achieved by moving the animal on a weighing scale or using an automatic walkover weighing system [7]. However, smaller farms cannot always afford these expensive, challenging, and costly to maintain investments [8]. Moreover, even if BW is measured at farms, the data often stay unshared, limiting its use by Dairy Herd Improvement (DHI) organizations. Previous studies showed the value of using observations from linear morphological classification in isolation [9,10], or in combination [11,12,13] in predicting BW, with accuracy ranging from 36 to 105 kg, and R² varying between 0.53 and 0.99. However, BW should be known regularly to monitor effectively, either at the farm or cow level. Therefore, accurate repeated measurements of morphological traits would be required, which could be costly and labor-intensive. Furthermore, linear morphological classification estimations are seldom taken during an animal’s lifetime [8,14], which would prevent controlling BW over time. 

To overcome some of these difficulties, researchers have developed automated methods making full use of computer vision techniques either through 2 [15,16], or 3-dimensional [17] devices. R² ranged from 0.75 to 0.98. More recently, Song et al. (2018) [18] obtained an RMSE of 41kg using a model whose predictors came from measurements estimated from a 3-D camera. Although a professional classifier’s intervention would no longer be required with such images, the devices may be expensive and require fine-tuning and maintenance, adding cost for the dairy. These devices are also cumbersome, as shown by their representations [16,17,18]. 

The current study aimed to expand on the range of BW prediction equations using mid-infrared (MIR) spectra. These data are routinely obtained (often every four weeks) on a large scale by DHI organizations. The advantages include the low cost of the routine collection, reproducibility of measurement from standardized, automated acquisition protocols for spectral data and animal characteristics, and the time saved by the operators who do not need to spend time on measurement. With many years of data stored by some DHI centers, a backward BW prediction is of interest for genetic purposes. The rationale is that milk composition assessed through milk MIR data can be linked to body condition changes associated with mobilization of fat reserves. A previous study featured a performance of Root Mean Square Error (RMSE) of cross-validation (CV) of 53 kg, and RMSE of prediction ranging between 37 to 64 kg for BW using MIR spectra, days in milk (DIM), parity, the month of test, and milk yield [8]. As the high number of predictors in a model can harm its robustness, notably for partial least squares (PLS) regression [19], reducing the number of spectral points to be considered in the predictive model from 1060 to 277 based on relevance in explaining the composition of milk (i.e., the regions between 950 and 1600, 1750 and 1800 cm^−1^ and between 2600 and 3000 cm^−1^) has been recommended [8]. 

In addition to validating the feasibility of predicting BW from the day in milk (DIM), parity, milk yield (MY), and milk MIR spectrum, this study also aimed to reduce the number of predictors further with the objective of removing irrelevant features [20]. The working hypotheses were (i) MIR spectral data are valuable for predicting BW together with DIM, parity, and MY, and (ii) multiple optimal models should coexist in predicting BW using MIR spectrum and animal characteristics. To better determine the performance of the approach, (i) we compared model performance, using MIR, parity, DIM, and MY with another using linear morphological traits predicting BW, and (ii) we interpreted the shape of the averaged large-scale predicted BW regarding the period of lactation. 

## 2. Materials and Methods

We used R software [21] for statistical analysis, the plsr function of the pls package [22] to calibrate the PLS regression models, and the caret [23] package as an interface to perform all model training and parametrization.

### 2.1. Training Data

The training dataset contained 1915 records collected between 2007 and 2016 made up of 360 Holstein cows from 9 herds (h01 to h09 in Table 1). A total of 1,161 records were collected from 93 Holstein cows (herds h03 and h06–h09) as part of the European Interreg Genotype plus Environment project (GplusE) (http://www.gpluse.eu, last visited 29 April 2021). Each sample included DIM, parity, test-day BW, MY, and milk MIR spectrum. Ragsdale (1934) reported that female Holstein reached a mature weight between 5 to 7 years [24]. Matthews and Fohrman (1954) exhibited similar observations [25]. Based on these considerations, the parity variable was divided into four classes (1st to 4th^+^), with 4th^+^ gathering cows within or beyond their fourth parity.

For herds h01 to h05, the MIR spectra were obtained using a MilkoScan FT6000 spectrometer (Foss Analytics, Hillerød, Denmark) or a Standard Lactoscope FT-MIR automatic (PerkinElmer, Waltham, MA, United States). Regarding the GplusE herds (h03, h06–h09), MIR spectral data formerly came from FT2 and FT6000 spectrometers (Foss Analytics, Hillerød, Denmark) or a Standard Lactoscope FT-MIR automatic (PerkinElmer, Waltham, MA, United States) [26]. Those spectra were then standardized according to the standardization procedure given in [27]. We applied a first derivative transformation with a gap of 5 wavelengths to the original spectra to flatten the spectral baseline drift [8]. Only spectral points covered by Foss, PerkinElmer, and Bentley instruments were kept to ensure the model transferability between instruments. Those were located from 925.66 cm^−1^ to 3995.78 cm^−1^ [27], representing 797 points. Moreover, another preselection of 277 spectral points was also possible [8]: [950 cm^−1^, 1600 cm^−1^], [1750 cm^−1^, 1800 cm^−1^], and [2600 cm^−1^, 3000 cm^−1^]. Consequently, we decided in this study to start the variables selection processes by considering two different starting points, one comprising all of the 797 initial spectral points, denoted by ALL, and the other having only the 277 points selected by empirical knowledge from Soyeurt et al. (2019) [8], subsequently expressed by HSO (for H. Soyeurt). Spectral records with a global H distance (GH), calculated using principal components explaining 99% of the spectral variability, higher than three were dropped [8,28,29].

The cows were weighed after milking using different walkover weighing pieces of equipment. A Fullwood branded system (Fullwood, Shropshire, UK) was used for herds h01–h03, for herd h04 Myscale Pro-W810 device (Gallagher, Canley, UK), an Autoweight (Dairymaster, Causeway, Co. Kerry, Ireland) system for h06–h09, and an incorporated scale gear inside an Astronaut A4 milking robot were employed for herds h05. Due to the variability of BW between age groups [24], 4 BW intervals were created and corresponded to the mean plus or minus the standard deviation of BW records calculated by parity class: (376,705), (449,767), (487,838), and (492,875) for cows in first to fourth+ parity, respectively. Records out of those ranges were discarded.

Finally, to ensure the consistency between BW and spectral records, a residual analysis based on predictions built from a partial least square (PLS) regression revealed other outliers. All samples with a residual value deviating by more than three standard deviations from the mean of all residuals were considered outliers and removed from the dataset. For each newly cleaned dataset, a new residual analysis was performed. This iterative procedure stopped when no more outliers were observed. The three cleaning steps (i.e., BW outliers, GH distances, and residual analysis) led to the deletion of 3.45% of the data, decreasing the number of records from 1915 to 1849, representing 360 different cows. Table 2 shows the descriptive statistics associated with this cleaned calibration dataset. All predictors were centered and scaled before modeling to give them the same importance in the regression.

### 2.2. Features Selection

Depending on the underlying induction model, the presence of irrelevant or redundant predictors could lead to performance degradation [20]. Therefore, we conducted variable selection for both datasets (i.e., HSO and ALL) to reduce the number of predictors. We used a univariate filter method, known to be tolerant in its selection [30]. This first technique, which did not consider the relationships between the variables, was a preselection step allowing only irrelevant predictors to be removed. Second, the recursive feature elimination (RFE) [31], based on the wrapper approach backward selection [32], enabled a more drastic selection by only picking a subset of the weakly relevant features.

#### 2.2.1. Filter Selection

For each dataset, each predictor X was successively used to calibrate a univariate generalized additive model (GAM) resumed as $BW=\beta_{0}+\beta_{1}\;s\left(X\right)+\varepsilon$, where s is the smoothing splines function indicating any functional relationship between X and BW [32], $\beta_{1}$, $\beta_{0}$ being respectively the model’s slope and intercept, and ε the error. The benefit of using a GAM model over a simple linear regression was that it could point out even non-linear relationships between the target and predictor variables [33]. For each fitted model, the null hypothesis (H0) asserts that no functional relationship exists between BW and X, which was tested through a nonparametric F statistic with a significance level set to 5%. Consequently, any model associated with a *p*-value lower than 5% means the rejection of H0, indicating the relevance of maintaining the predictor (X) in the dataset. At the end of that selection step, two new datasets with fewer predictors than the former ones arose, namely ALL_SBF and HSO_SBF, with SBF standing for “selected by filter” (Table 3).

#### 2.2.2. Wrapper Selection

A wrapper is a multivariate selection method involving the induction algorithm in the variable evaluation mechanism [20,34], thus adding consistency in the variables’ ranking regarding the final objective. The wrapper method chosen was RFE, which was composed of four steps. First, the predictors were ranked in descending order according to the relevance to predict BW. We used variable importance in the projection (VIP) and BETA scores on the entire training dataset to assess the relevance to predict BW [35]. Second, we split each ranked set of features (i.e., using VIP or BETA) into subsets. The number of subsets depended on the number of features in the ranked set. To limit the computational cost, we did not use all combinations of ranked features. To be clear, the first subset encompassed the total number of features (Nf), while those following included Nf-gap features. The different values taken by the variable gap depended on the number of features remaining (i.e., gap = 5 for (Nf, 255), gap = 2 for (252,200), and gap = 1 for (199,2) features). When a subset was created, a PLSR was built to re-rank the features following their information relevancy as explained previously and to evaluate the model by calculating a stratified 10-fold cross-validation RMSE (RMSEscv), as recommended by Kohavi (1995) [36]. For the stratification, we defined 4 BW intervals based on the minimum, maximum BW values and the first three quantiles: (428,564), (564,614), (614,671), and (671,820). Each cross-validation fold contained the same number of records in each BW interval. The wrapper selection was conducted on HSO, ALL_SBF, and HSO_SBF. Therefore, a total of 1,280 different subsets of features were created, as described in Table 3. In order to limit the computational cost, by still focusing on the most generalizable and parsimonious models, we kept for the further validation procedures, only the 13 models including an equal or lower number of features compared to the model having the lowest RMSE_SCV_ + (tolerance/100) * lowest RMSE_SCV_ where the tolerance ranged from 0 to 12. Consequently, we ended with a total of 78 subsets (i.e., each set ALL_SBF_VIP, ALL_SBF_BETA, HSO_VIP, HSO_BETA, HSO_SBF_VIP, and HSO_SBF_BETA contained 13 subsets of predictors).

### 2.3. Herd Independent Internal Validation

The stratified cross-validation, as proposed in this study, could have led to over-fitting of the models. Therefore, a herd-independent internal validation (iv) was used to define the optimal number of PLS factors and to identify the best subsets of features. The creation of the folds for this validation respected the following rules: herds in the calibration set must not be in the validation set, and the validation set could contain more than one herd to have a validation size comprised between 10 and 30% of the initial dataset. The partitioning was repeated 101 times for the 78 previously selected subsets. To make these models parsimonious, we decided to limit the maximum number of PLS factors to 10. This procedure was done for the 78 selected subsets of features.

Consequently, 7878 PLS models were built. For each kind of model, we computed the average herd-independent RMSE ($\bar{RMSE_{iv}}$) and the associated standard deviation ($\bar{RMSESD_{iv}}$). Then, we performed 10-fold stratified cross-validations on the same 7878 models to evaluate the gap between herd-independent and stratified cross-validation performance. To do so, we calculated the average RMSE per kind of models ($\bar{RMSE_{SCV}}$) and its standard deviation ($\bar{RMSESD_{SCV}}$). Ultimately, we selected the six best models (one per kind of model) based on these two averages RMSE and their respective standard deviation.

### 2.4. External Validation

The external validation dataset included 4067 records collected from 231 Australian Holstein cows between October 2015 and December 2017 at the Ellinbank Research Farm belonging to Agriculture Victoria Research (h10 in Table 1). A walkover scale DeLaval Automatic Weigh System AWS100 (DeLaval International, Tumba, Sweden) was used for herd h10 [37] to measure test-day BW. The MIR spectra were provided by a spectrometer model 2000 (Bentley Instruments, Chaska, MN, USA). We approximated the standardization of the spectra by applying the technique developed by Grelet et al. (2015) [27] but using a deferred standardization table. 

Before processing the external validation, we calibrated the six selected final models over the entire training dataset (N = 1849 records). Their external validation performance was assessed by calculating the RMSE of validation (RMSE_v_) on this fully independent h10 dataset.

### 2.5. Bodyweight Prediction from DHI Dataset

Ultimately, we applied the models offering the best external validation performance to the DHI dataset managed by the Walloon Breeding Association (Awé, Ciney, Belgium), comprising 3,769,477 records collected from 276,728 Holstein cows, with The MIR spectral points provided by FT6000 and FT+ spectrometers (Foss Analytics, Hillerød, Denmark). Although no observed reference BW measurements were present in this dataset, the idea was to verify if the average trends of predicted BW within and across lactations were those expected. As a link exists between BW and milk composition, we compared the average predicted BW’s evolution to the observed milk production and predicted fatty acids curves. The fatty acids content was predicted using the equations developed by Soyeurt et al. (2011) [38]. 

## 3. Results and Discussion

### 3.1. Features Selection

The role of the selection algorithm was to maintain spectral points that had useful information and sufficient variability and eliminate spectral areas containing noises. A first bare selection consisted of picking the most relevant features by using SBF. From the results presented in Table 3, this selection reduced variables number of ALL by 52.62% and HSO by 43.21%. For both datasets, the parity, DIM, and MY were still present. The selection made by Soyeurt et al. (2019) [8] based on previous knowledge allowed a consistent skimming on irrelevant predictors. Indeed, the areas excluded from HSO were also excluded from ALL at 57.61% using SBF. 

The wrapper selection enabled a more drastic selection by only picking a subset of the weakly relevant features from the analysis of 1280 different subsets (Table 3). To limit the computational cost and to ensure the use of a parsimonious model, we selected the 13 models for which the RMSE_SCV_ were equal to the lowest observed RMSE_SCV_ + (tolerance/100) * lowest observed RMSE_SCV_ with a tolerance ranging from 0 to 12. The models associated with the lowest RMSE_SCV_ included the maximum number of informative features, ranging from 141 to 282 features (Table 3). It was interesting to notice that the HSO_BETA conserved all HSO features, confirming the interest of the variables selected by experience.

### 3.2. Herd-Independent Internal Validation

**Figure 1:**
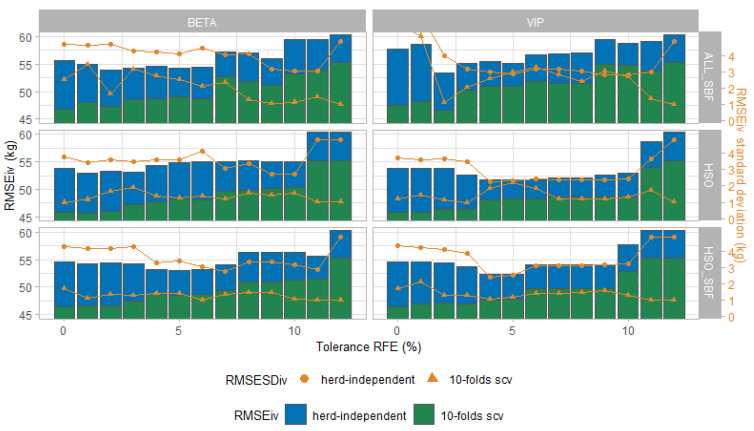
Root mean squared error for herd independent validation repeated 101 times (RMSE_iv_) and its standard deviation from the 13 models selected from the recursive feature elimination (RFE) tolerance. RMSE for the stratified 10-fold cross-validation (RMSE_SCV_) for the same models and its standard deviation are also plotted.

For each of the 13 selected models (i.e., one model per tolerance value ranging from 0 to 12), we performed a herd independent internal validation repeated 101 times. Figure 1 summarizes the results by profile and dataset for each tolerance value. $\bar{RMSE_{iv}}$ ranged between 51.6 ± 2.25 and 60.3 ± 4.83 kg. HSO_VIP did the best with a $\bar{RMSE_{iv}}$ of 53.7 ± 3.03 kg. Regardless of the profile, ALL_SBF was the worst performer dataset with $\bar{RMSE_{iv}}$ of 56.7 ± 3.95. These results showed how relevant was the preselection made by Soyeurt et al. (2019) [8]. By taking into account $\bar{RMSE_{iv}}$, $\bar{RMSESD_{iv}}$, $\bar{RMSE_{SCV}}$, and $\bar{RMSESD_{SCV}}$ in Figure 1, six models can be kept for the external validation. Their performance results are summarized in Table 4. The best number of features to optimize $\bar{RMSE_{iv}}$ and $\bar{RMSE_{SCV}}$ were bound between 8 to 20, associated with HSO_VIP, HSO_SBF_BETA, and HSO_SBF_VIP. 

Before performing the Australian data’s external validation (h10, Table 2), we build the six selected models in Table 4 using the entire calibration dataset (N = 1849 records). RMSE_SCV_ ranged from 48 to 52 kg, which was the range of accuracy mentioned by Soyeurt et al. (2019) [8]. Therefore, the current study confirmed the feasibility of predicting an indicator of BW using milk MIR spectrum, MY, DIM, and parity.

### 3.3. External Validation

From Table 4, it can be seen that these six selected models did not always match with the best externally performing models in light of results obtained in the external validation. One of the probable causes could be that the standardization of the validation set (h10) was only approached via a standardization table remote in time, suggesting that some spectral zones would be less sensitive to artifacts and, consequently, to standardization. However, some of these models performed reasonably well in external validation, with good RMSE_v_ values ranging from 52 to 56 kg (Table 4). 

### 3.4. Model Interpretation

The remainder of the analysis focused on the three models with the best RMSEv statistics (52 and 56 kg): HSO_VIP, HSO_BETA, and HSO_SBF_BETA. The obtained models using those subsets of features were quite similar. HSO_VIP kept 11 features distributed into 3 PLS components. HSO_BETA involved 7 features for 3 components, where HSO_SBF_BETA contained 8 features summarized in 7 PLS components (Table 4). All subsets of features had the MY, DIM, and parity in common but had selected different spectral points. Despite some slight disparities between RMSEv values of those three models (52 vs. 56 kg), the predictions given by those three models were highly correlated (R > 95%). Consequently, even if the predictors selected were different for these models, their respective combinations within the corresponding PLS model brought the same level of usefulness in predicting BW. The difference occurred at the spectral level, which would be explained by the high correlations between adjacent spectral points. Therefore, neighboring spectral points shared similar information. 

**Figure 2:**
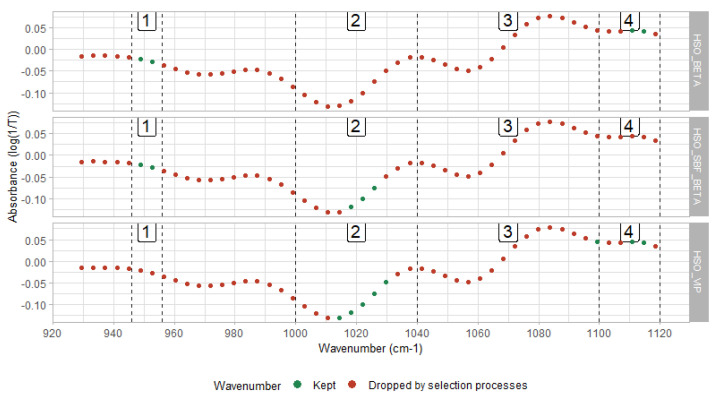
Spectral representation of the 3 models providing the best RMSE of validation.

Figure 2 shows that the spectral zones of interest bounded by the interval (926 cm^−1^, 1120 cm^−1^), which is part of the so-called fingerprint region (926 cm^−1^, 1500 cm^−1^) [39]. Moreover, this zone relates to a high heritability value, meaning that transmission is made across generations and confirms that this spectral area was not noisy [40]. The absorbance of the spectral regions between 900 and 1153 cm^−1^ could be explained by the C–O and C–C stretching modes [39], related to the chemical structure of several major milk components such as fat, sugar, and protein [40]. Lactose association could be extended to the spectral ranges (950 cm^−1^, 1200 cm^−1^), stressing that the interval (1000 cm^−1^, 1150 cm^−1^) was related to lactic acid (lactate) [41]. Conclusively, the region of interest (926 cm^−1^, 1200 cm^−1^) could be stated as the lactose-region [42].

Figure 2 shows how closely some spectral points were gathered when involved in a prediction model. The first cluster included the spectral points at 949 cm^−1^ and 953 cm^−1^. This zone (946 cm^−1^, 956 cm^−1^) was activated twice by the three PLS models. The interval (1000 cm^−1^, 1040 cm^−1^) corresponded to the second grouping area whose spectral points were attributed to lactate [43]. This zone had the largest number of activated points when involved in BW prediction. The third zone included points in (1040 cm^−1^, 1100 cm^−1^). This area was mainly associated with lactose measurement [43]. The fourth zone, such as the second zone, was also connected to lactate. Lactic acid showed absorption bands, notably at 1115 cm^−1^ [41], which corresponds to one of the points activated when this zone was involved in the modeling. Therefore, a spectral zone was then defined by a set of contiguous and delimited spectral points providing useful information explaining BW’s variance. All three selected models used a various mix of these spectral zones in different proportions and combinations. This selection depended on (i) the initial subset of predictors and (ii) the ranking function associated with RFE.

Figure 2 points out that the spectral areas activated were quite different for the same ranking function (HSO_BETA vs. HSO_SBF_BETA), thus suggesting zones 2 and 4 as reciprocal substitutes. Furthermore, through HSO_VIP and HSO_BETA, Figure 2 shows that the two ranking methods can order the most important variables differently from the same initial subset of predictors. The dataset’s particularity that adjacent variables were highly correlated may have played a crucial role in that discrepancy between ranking profiles. Zone 4 operated as a pivot for the substitutions between zone 1 and zones 2 and 3. This discussion should be put into perspective because the standardization of the outer-validation dataset (h10) was not perfect, and the analyses to be made on an external validation with a standardization carried out in better circumstances may be different.

Nevertheless, it was clear that there is no one-of-a-kind best model. There are instead many models, each of them looking for relevant information in distinct spectral regions. This claim is in line with the analysis carried out by Kohavi and John (1997) [20], who showed that some weakly relevant predictors might coexist in the sense that, although providing useful information to the model, they could be interchanged without affecting its predictive quality.

Besides, it appeared that spectral information brought to the predictive model was useful and relevant when associated with the variables MY, DIM, and parity in BW prediction. The added value of using spectral points was quantified by comparing the three aforementioned models with another, including DIM, MY, and parity features. This model with three features for two PLS components had an RMSEv of 70 kg against 52 kg for the best results obtained using the spectral data as additional features. 

### 3.5. Implementation of BW Predictive Models on DHI Database

**Figure 3:**
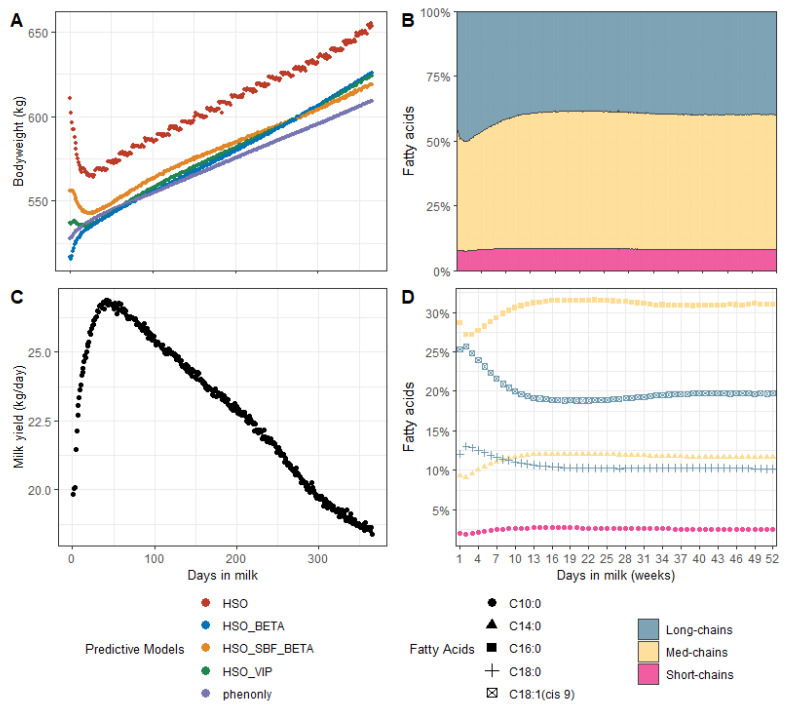
Evolution of the predicted bodyweight (**A**), the predicted content of some fatty acids groups in fat (**B**), the milk yield (**C**), and the predicted content of some individual fatty acids in fat (**D**). HSO is the shortcut that subsets of features used by Soyeurt et al. (2019), while phenonly stands for a subset containing only milk yield, parity and DIM.

We applied the best models on the Walloon DHI dataset. Figure 3A shows the behavior of the different BW predictions’ averages, computed by parity and DIM on this DHI dataset. For the sake of clarity, only the first parity is given, while the other parity showed similar results. It could be observed that the average predicted BW evolutions across lactation revealed the expected behavior, especially the BW loss in early lactation caused by the concurrent effects of energy demand increase for milk production (Figure 3C) and feed intake partially prevented due to appetite drop and physical limitation in rumen capacity [44]. The contrast of BW evolutions between HSO, HSO_BETA, and other plotted models was remarkable. Dettmann et al. (2020) showed similar weight curves for Holstein cows to what was observed through the HSO_SBF_BETA equation, both in behavior and size [45].

Furthermore, changes occur in milk fat composition in early lactation [46]. As the milk fatty acids content can be predicted by MIR technology [38], we assumed that indicators of their variations would also be present in the MIR spectral data. To that end, we predicted the milk FA content of the DHI records from milk MIR spectra and then averaged by DIM and parity. Figure 3B and D respectively show the evolution of FA groups’ content in fat (i.e., short, medium, and long-chain FAs) and the FA drivers of change (C10:0, C14:0, C16:0, C18:0, and C18:1 cis-9 expressed in fat) within lactation. We were interested in measuring the linear correlation between each MIR spectral point kept as predictors by the HSO_SBF_BETA, HSO_BETA, HSO_VIP models, and the aforementioned FA. Figure 2 highlights those points of interest in green arranged in four spectral zones. The analysis showed that C10:0 was moderately correlated with the spectral points at 948.81 cm^−1^, and 952.66 cm^−1^ (zone1), 1029.80 cm^−1^ (zone2), and 1110.80 cm^−1^ (zone4) with a respective correlation of −0.55, −0.56, −0.42, and 0.63. The medium-chain FAs were weakly to moderately correlated with points in the first, second, and third spectral zones, featuring correlations of −0.70, −0.67, −0.38, and 0.53 for the points at 948.81 cm^−1^, and 952.66 cm^−1^ (zone1), 1029.80 cm^−1^ (zone2), and at 1110.80 cm^−1^ (zone3), respectively. The long-chain FAs happened to be moderately correlated with points belonging to the first zone (948.81 cm^−1^ and 952.66 cm^−1^ resp. −0.61, −0.51) and weakly correlated with the point at 1114.65 cm^−1^ located in zone 4 (−0.24).

Knowledge about FA content and their evolution through lactation could be a differentiator for BW prediction. Although their daily changes are not explicitly found in the MIR, predictive models may find signals in the spectral data that explain some aspects of the animal BW. This kind of a posteriori analysis is valuable for model validation. Although Table 4 shows that three models stood out, we found out that the average predicted BW evolution model HSO_BETA exhibited a completely unexpected behavior since it tends to underestimate the average predicted BW in the very early lactation in comparison with the other models. While the external validation showed similar performance for the models HSO_VIP and HSO_SBF_BETA (RMSEv = 51 kg), we favor HSO_SBF_BETA, with a trend of averaged predicted BW across lactation that is consistent with the literature. Nevertheless, a predictive model is dependent on the quality and representativeness of its training data. Therefore, these models may need to evolve in light of the emergence of new data that may highlight other MIR spectral predictors’ relevancy in the future. 

In the calibration dataset, 39 records collected from 39 different cows belonging to the h02 herd (Walloon Breeding Association, Belgium, May-07) had morphological information. We used those records to predict BW using linear morphological classification proposed by Saussez (2017) [47] for the Walloon Breeding Association. The obtained RMSE was 71 kg for the equation using linear classification vs. 65, 60, and 54 kg for HSO_BETA, HSO_VIP, HSO_SBF_BETA, respectively. These results highlighted a positive interest in developing prediction models using MIR data. Although those models’ predictions cannot replace the actual BW measurements, they can be considered an indicator that smoothed out the measurement’s variability. Besides, thanks to a large amount of repeated data and the homogeneity of the phenotypes needed to perform the BW prediction proposed in this study, the predicted BW phenotypes are expected to be easily comparable between cows and herds, providing relevant information for breeding and management.

Furthermore, DHI organizations could potentially provide these predictions as a new service. The development of a multi-trait approach combining linear morphological classification, actual BW measurement, and MIR predicted BW could improve BW’s accuracy. The repeated measurements of MIR predicted BW for the same cows on a routine basis would also define BW changes that can be interesting in improving dairy cows’ feed efficiency.

## 4. Conclusions

Although multiple optimal models coexisted in predicting BW using MIR spectra, it was shown that the usage of these spectra within a modeling process to predict BW is relevant, and the performance achieved were comparable with such offered by methods using linear morphological traits as input variables. However, the advantage of the method proposed in this paper is to obtain highly reproducible BW predictions obtained at low cost and every 4–6 weeks for the productive cows participating in the routine milk recording. Moreover, thanks to the predictive model’s features, it is possible to rapidly operate a past BW prediction to obtain a large-scale database for genetic purposes. 

## Figures and Tables

**Table 1:** Sampling information for each studied herd included in the study.

Herd	Origin	Country	Std ^1^	Period Coverage		N (n) ^2^
				Min	Max	
h01	Walloon Breeding Association	Belgium	No	Oct-08	Dec-08	32 (21)
h02	Walloon Breeding Association	Belgium	No	May-07	May-07	41 (41)
h03	Walloon Agricultural Research Center	Belgium	No	Feb-11	Sept-12	130 (29)
h03	Walloon Agricultural Research Center	Belgium	Yes	Jan-15	Oct-15	23 (14)
h04	University of Alberta	Canada	No	Jul-14	Dec-14	396 (132)
h05	University of Liège	Belgium	No	May-14	Dec-14	155 (47)
h06	The Agri-Food and Biosciences Institute	Ireland	Yes	Sept-14	Dec-14	188 (31)
h07	Aarhus University	Denmark	Yes	Oct-14	Jan-15	635 (18)
h08	Leibniz Institute for Farm Animal Biology	Germany	Yes	May-15	Jun-16	180 (12)
h09	University College Dublin	Ireland	Yes	Feb-15	May-15	135 (18)
					Total	1915 (360) **^3^**
h10	Agriculture Victoria Research	Australia	No	Oct-15	Dec-17	4067 (231)

^1^ If the spectral data were standardized, the Std column was set to “yes”. ^2^ N = number of records; n = number of cows. ^3^ The total number of cows (360) does not coincide with the sum of the number of cows of each row because the standardized h03 (fourth row) and the not standardized h03 (third row) share 3 cows in common.

**Table 2:** Descriptive statistics of training and validation datasets.

Variables ^1^	Measure ^2^	Training ^3^									Validation ^4^
		h01	h02	h03	h04	h05	h06	h07	h08	h09	h10
Total number of records		31	39	146	377	153	174	630	174	125	4066
Parity	Primiparous	4	14	32	127	46	45	213	12	0	963
	Multiparous	27	25	114	250	107	129	417	162	125	3103
Bodyweight	Mean	677	675	624	628	622	614	599	607	656	550
	s.d.	70	73	63	65	75	80	86	60	54	65
Milk yield	Mean	22	29	23	20	20	31	38	41	35	26
	s.d.	8	10	8	5	7	11	10	7	7	5
DIM	Min	53	15	5	3	1	5	4	5	6	37
	Q1	101	76	58	59	87	16	18	18.3	22	95
	Median	166	144	134	116	170	28	28	29.5	31	105
	Q3	210	212	210	181	265	39	40	39	40	116
	Max	280	475	424	312	512	50	50	50	50	161

^1^ Variables in the datasets. DIM = days in milk. ^2^ Function applied to a variable or an entire dataset, per herd. Total number of records (total); number of records belonging to primiparous/multiparous cows (primiparous/multiparous); average value of a specific variable (mean); standard deviation of a specific variable (s.d.); minimum or maximum (min/max); first, second, or third quartile (Q1, median, Q3). ^3^ Statistics for the datasets used for calibration (h01–h09). ^4^ Statistics for the dataset used for validation (h10).

**Table 3:** Subsets and number of selected features for the selection by filter (SBF) and the recursive feature elimination (RFE). Performance of the best models selected by RFE.

Subset ^1^	Nfeat. ^2^	SBF Selection		RFE Selection			
		Subset ^3^	Nfeat. ^2^	Subset ^4^	Nsubsets ^5^	max. Nfeat. ^6^	RMSE_SCV_ ^7^
ALL	800	ALL_SBF	379	ALL_SBF_VIP	251	282	47 ± 1.29
				ALL_SBF_BETA	251	204	47 ± 1.37
HSO	280	HSO_SBF	159	HSO_SBF_VIP	158	141	47 ± 1.40
				HSO_SBF_BETA	158	143	47 ± 1.45
		/	/	HSO_VIP	231	244	46 ± 1.30
				HSO_BETA	231	280	46 ± 1.40

^1^ Original subsets of predictors before any selection step; All the mid-infrared spectral points originally considered (ALL); Only the mid-infrared spectral points at wavenumbers (950 cm^−1^, 1600 cm^−1^), (1750 cm^−1^, 1800 cm^−1^), and (2600 cm^−1^, 3000 cm^−1^) originally considered before selection steps (HSO). ^2^ Corresponding number of features to the subset. ^3^ Subsets of predictors after the first selection step, i.e., selection by filter (SBF). ^4^ Subsets of predictors after the recursive feature elimination (RFE) selection step; SBF means that a first selection was made before RFE using selection by filter; VIP (variables in projection) and BETA (beta coefficients) refers to the ranking methods chosen for RFE. ^5^ Number of created subsets by the recursive feature elimination selection step. ^6^ Maximum number of selected features. ^7^ Stratified 10-fold cross-validation root mean squared error (kg).

**Table 4:** Prediction performance of the six best models per kind of subsets.

Subset ^1^	Nfeatures ^2^	Herd Independent Internal Validation		Calibration from Entire Dataset			RMSE_v_ ^7^
		RMSE_iv_ ^3^	RMSE_SCV_ ^4^	Ncomp ^5^	RMSE_SCV_ ^4^	R²_scv_ ^6^	
		(kg)	(kg)		(kg)		(kg)
ALL_SBF_BETA	5	56 ± 3.16	51 ± 1.07	2	52 ± 1.64	0.54	101
ALL_SBF_VIP	62	53 ± 3.98	47 ± 1.13	5	48 ± 1.54	0.61	130
HSO_BETA	7	55 ± 2.71	50 ± 1.46	3	51 ± 1.79	0.56	56
HSO_VIP	11	52 ± 2.34	49 ± 1.22	3	50 ± 1.63	0.58	52
HSO_SBF_BETA	8	53 ± 3.06	49 ± 1.00	7	50 ± 1.51	0.58	52
HSO_SBF_VIP	20	52 ± 2.38	48 ± 1.06	7	48 ± 1.56	0.6	116

^1^ Subsets of predictors after the recursive feature elimination (RFE) selection step; SBF means that a first selection was made before RFE using selection by filter; VIP (variables in projection) and BETA (beta coefficients) refers to the ranking methods chosen for RFE; ALL means that originally all the mid-infrared spectral points were considered; HSO means that only the mid-infrared spectral points at wavenumbers (950 cm^−1^, 1600 cm^−1^), (1750 cm^−1^, 1800 cm^−1^), and (2600 cm^−1^, 3000 cm^−1^) were initially considered before selection steps. ^2^ Corresponding number of features to the subset. ^3^ Root mean squared error from the herd-independent validation repeated 101 times. ^4^ Root mean squared error of 10-folds stratified cross-validation. ^5^ Number of latent variables kept by the partial least square regression. ^6^ Coefficient of determination. ^7^ Root mean squared error of validation using Australian dataset.

## Data Availability

Restrictions apply to the availability of these data. Data were obtained from the Walloon Breeding Association (Ciney, Belgium), the Walloon Agricultural Research Center (Gembloux, Belgium), the Dairy Research and Technology Center (DRTC) of the University of Alberta (Alberta, Canada), the University of Liège (Liège, Belgium), the Ellinbank Research Farm belonging to Agriculture Victoria Research (Australia), and from the Genotype Plus Environment (GplusE) Project, namely, data from The Agri-Food and Biosciences Institute (Ireland), the Aarhus University (Denmark), the Leibniz Institute for Farm Animal Biology (Germany), the Walloon Agricultural Research Center (Gembloux, Belgium) and the University College Dublin (Ireland). They are available from the authors with the permission of the related aforementioned third parties.

## References

[1] Thorup V.M., Edwards D., Friggens N.C. (2012). On-farm estimation of energy balance in dairy cows using only frequent body weight measurements and body condition score. J. Dairy Sci..

[2] Berry D.P., Lee J.M., Macdonald K.A., Stafford K., Matthews L., Roche J.R. (2007). Associations Among Body Condition Score, Body Weight, Somatic Cell Count, and Clinical Mastitis in Seasonally Calving Dairy Cattle. J. Dairy Sci..

[3] National Research Council (2001). Nutrient Requirements of Dairy Cattle.

[4] Blaxter K.L., Clapperton J.L. (1965). Prediction of the amount of methane produced by ruminants. Br. J. Nutr..

[5] Herd R.M., Arthur P.F., Donoghue K.A., Bird S.H., Bird-Gardiner T., Hegarty R.S. (2014). Measures of methane production and their phenotypic relationships with dry matter intake, growth, and body composition traits in beef cattle. J. Anim. Sci..

[6] van Straten M., Shpigel N.Y., Friger M. (2009). Associations among patterns in daily body weight, body condition scoring, and reproductive performance in high-producing dairy cows. J. Dairy Sci..

[7] Alawneh J.I., Stevenson M.A., Williamson N.B., Lopez-Villalobos N., Otley T. (2011). Automatic recording of daily walkover liveweight of dairy cattle at pasture in the first 100 days in milk. J. Dairy Sci..

[8] Soyeurt H., Froidmont E., Dufrasne I., Hailemariam D., Wang Z., Bertozzi C., Colinet F.G., Dehareng F., Gengler N. (2019). Contribution of milk mid-infrared spectrum to improve the accuracy of test-day body weight predicted from stage, lactation number, month of test and milk yield. Livest. Sci..

[9] Heinrichs A.J., Rogers G.W., Cooper J.B. (1992). Predicting Body Weight and Wither Height in Holstein Heifers Using Body Measurements. J. Dairy Sci..

[10] Heinrichs A.J., Heinrichs B.S., Jones C.M., Erickson P.S., Kalscheur K.F., Nennich T.D., Heins B.J., Cardoso F.C. (2017). Short communication: Verifying Holstein heifer heart girth to body weight prediction equations. J. Dairy Sci..

[11] Enevoldsen C., Kristensen T. (1997). Estimation of Body Weight from Body Size Measurements and Body Condition Scores in Dairy Cows. J. Dairy Sci..

[12] Banos G., Coffey M.P. (2012). Technical note: Prediction of liveweight from linear conformation traits in dairy cattle. J. Dairy Sci..

[13] Haile-Mariam M., Gonzalez-Recio O., Pryce J.E. (2014). Prediction of liveweight of cows from type traits and its relationship with production and fitness traits. J. Dairy Sci..

[14] Vanrobays M.-L., Vandenplas J., Hammami H., Froidmont E., Gengler N. (2015). Short communication: Novel method to predict body weight of primiparous dairy cows throughout the lactation. J. Dairy Sci..

[15] Stajnko D., Brus M., Hočevar M. (2008). Estimation of bull live weight through thermographically measured body dimensions. Comput. Electron. Agric..

[16] Tasdemir S., Urkmez A., Inal S. (2011). Determination of body measurements on the Holstein cows using digital image analysis and estimation of live weight with regression analysis. Comput. Electron. Agric..

[17] Salau J., Haas J.H., Junge W., Thaller G. (2016). Extrinsic calibration of a multi-Kinect camera scanning passage for measuring functional traits in dairy cows. Biosyst. Eng..

[18] Song X., Bokkers E.A.M., van der Tol P.P.J., Koerkamp P.W.G.G., van Mourik S. (2018). Automated body weight prediction of dairy cows using 3-dimensional vision. J. Dairy Sci..

[19] Nadler B., Coifman R.R. (2005). The prediction error in CLS and PLS: The importance of feature selection prior to multivariate calibration. J. Chemom..

[20] Kohavi R., John G.H. (1997). Wrappers for feature subset selection. Artif. Intell..

[21] R Core Team (2020). R: A Language and Environment for Statistical Computing.

[22] Bjørn-Helge M., Wehrens R., Liland K.H. (2019). Pls: Partial Least Squares and Principal Component Regression; R Package Version 2.7-2.

[23] Kuhn M. (2020). Caret: Classification and Regression Training; R Package Version 6.0-86.

[24] Ragsdale A.C. (1934). Growth standards for dairy cattle. Missouri Agric. Exp. Sin. Bull..

[25] Matthews C.A., Fohrman M.H. (1954). Beltsville Growth Standards for Holstein Cattle.

[26] Grelet C., Froidmont E., Foldager L., Salavati M., Hostens M., Ferris C.P., Ingvartsen K.L., Crowe M.A., Sorensen M.T., Pierna J.F. (2020). Potential of milk mid-infrared spectra to predict nitrogen use efficiency of individual dairy cows in early lactation. J. Dairy Sci..

[27] Grelet C., Pierna J.A.F., Dardenne P., Baeten V., Dehareng F. (2015). Standardization of milk mid-infrared spectra from a European dairy network. J. Dairy Sci..

[28] Garrido-Varo A., Garcia-Olmo J., Fearn T. (2019). A note on Mahalanobis and related distance measures in WinISI and The Unscrambler. J. Near. Infrared Spectrosc..

[29] Delhez P., Ho P.N., Gengler N., Soyeurt H., Pryce J.E. (2020). Diagnosing the pregnancy status of dairy cows: How useful is milk mid-infrared spectroscopy?. J. Dairy Sci..

[30] Kuhn M., Johnson K., Kuhn M., Johnson K. (2013). An Introduction to Feature Selection. Applied Predictive Modeling.

[31] Guyon I., Weston J., Barnhill S., Vapnik V. (2002). Gene Selection for Cancer Classification using Support Vector Machines. Mach. Learn..

[32] Hastie T., Tibshirani R. (1986). Generalized Additive Models. Stat. Sci..

[33] Vislocky R.L., Fritsch J.M. (1995). Generalized Additive Models versus Linear Regression in Generating Probabilistic MOS Forecasts of Aviation Weather Parameters. Weather Forecast..

[34] John G.H., Kohavi R., Pfleger K. Irrelevant Features and the Subset Selection Problem. Proceedings of the Eleventh International Conference.

[35] Chong I.-G., Jun C.-H. (2005). Performance of some variable selection methods when multicollinearity is present. Chemometr. Intell. Lab. Syst..

[36] Kohavi R. A study of cross-validation and bootstrap for accuracy estimation and model selection. Proceedings of the 14th International Joint Conference on Artificial Intelligence-Volume 2.

[37] Ho P.N., Marett L.C., Wales W.J., Axford M., Oakes E.M., Pryce J.E. (2020). Predicting milk fatty acids and energy balance of dairy cows in Australia using milk mid-infrared spectroscopy. Anim. Prod. Sci..

[38] Bastin C., Gengler N., Soyeurt H. (2011). Phenotypic and genetic variability of production traits and milk fatty acid contents across days in milk for Walloon Holstein first-parity cows. J. Dairy Sci..

[39] Karoui R., Mouazen A.M., Dufour E., Pillonel L., Schaller E., Picque D., De Baerdemaeker J., Bosset J.O. (2006). A comparison and joint use of NIR and MIR spectroscopic methods for the determination of some parameters in European Emmental cheese. Eur. Food Res. Technol..

[40] Soyeurt H., Misztal I., Gengler N. (2010). Genetic variability of milk components based on mid-infrared spectral data. J. Dairy Sci..

[41] Martín-del-Campo S.T., Picque D., Cosío-Ramírez R., Corrieu G. (2007). Evaluation of Chemical Parameters in Soft Mold-Ripened Cheese During Ripening by Mid-Infrared Spectroscopy. J. Dairy Sci..

[42] Zaalberg R.M., Shetty N., Janss L., Buitenhuis A.J. (2019). Genetic analysis of Fourier transform infrared milk spectra in Danish Holstein and Danish Jersey. J. Dairy Sci..

[43] Picque D., Lefier D., Grappin R., Corrieu G. (1993). Monitoring of fermentation by infrared spectrometry: Alcoholic and lactic fermentations. Anal. Chim. Acta.

[44] Walsh S.W., Williams E.J., Evans A.C.O. (2011). A review of the causes of poor fertility in high milk producing dairy cows. Anim. Reprod. Sci..

[45] Dettmann F., Warner D., Buitenhuis B., Kargo M., Kjeldsen A.M.H., Nielsen N.H., Lefebvre D.M., Santschi D.E. (2020). Fatty Acid Profiles from Routine Milk Recording as a Decision Tool for Body Weight Change of Dairy Cows after Calving. Animals.

[46] Gross J., van Dorland H.A., Bruckmaier R.M., Schwarz F.J. (2011). Milk fatty acid profile related to energy balance in dairy cows. J. Dairy Res..

[47] Saussez G. (2017). Contribution à L’étude de L’efficience Énergétique des Vaches Laitières en Wallonie.

